# Outcomes of Staphylococcal Prosthetic Joint Infection After Hip Hemiarthroplasty: Single Center Retrospective Study

**DOI:** 10.3390/medicina61040602

**Published:** 2025-03-26

**Authors:** Ahmed Nageeb Mahmoud, Nicholas R. Brule, Michael Suk, Daniel Scott Horwitz

**Affiliations:** 1 Geisinger Medical Center, Danville, PA 17821, USA; nbrule@geisinger.edu (N.R.B.); msuk@geisinger.edu (M.S.); 2 Faculty of Medicine, Ain Shams University, Cairo 11517, Egypt

**Keywords:** *Staphylococcus*, Staphylococcal infections, infections, periprosthetic infections, prosthetic joint infection, hemiarthroplasty, hip fractures

## Abstract

*Background and Objectives*: When prosthetic joint infections, known for their high morbidity, are caused by high-virulence organisms such as *Staphylococcus*, the outcomes are even worse. This study aims to examine the outcomes of staphylococcal prosthetic joint infections after hemiarthroplasty for hip trauma patients, which has not been particularly reported. *Materials and Methods*: A retrospective study was performed in a level 1 trauma center to review all the cases of prosthetic joint infections in our database. Patients’ demographics, clinical inpatient, surgical, and outpatient notes, laboratory results, and serial radiographs were assessed to extract all relevant data. *Results*: Out of 2477 hip hemiarthroplasty cases reviewed, a total of 36 prosthetic joint infection cases caused by *Staphylococcus* species in 36 patients were included in this study. Patients were 26 females and 10 males with a mean age of 76.5 years at the time of surgery. Fifteen cases had infections with methicillin-resistant *Staphylococcus aureus* (MRSA) while twenty-one cases had infections with other *Staphylococcus* species. The mean follow-up for all cases is 43.5 months. Twenty-nine cases underwent at least a single trial for surgical debridement and implant retention surgery, and only nine (31%) had successful debridement and implant retention. The 3-month, 1-year, and 3-year cumulative mortality for all cases was 22.2, 30.5, and 41.7%, respectively. *Conclusions*: Staphylococcal hemiarthroplasty infection is a devastating complication that is associated with a low success (31%) of implant retention surgery, significantly high morbidity, and high cumulative mortality.

## 1. Introduction

Hip hemiarthroplasty (HA) is a widely utilized treatment option for displaced femoral neck fractures in the elderly [1,2]. We recognize prosthetic joint infection (PJI), after arthroplasty as a devastating complication that is associated with high morbidity and mortality [3,4]. Importantly, *Staphylococcus* is the causative organism in up to approximately two-thirds of PJI cases [5].

The available treatment options for PJI fall into two broad categories, namely debridement, antibiotics, and implant retention (DAIR) [6,7,8,9,10] or implant removal (IRm) with or without secondary surgery [11,12,13]. However, the available studies regarding staphylococcal PJI [6,7,8,9,10,11,12,13] included both hemiarthroplasty and total joint arthroplasty. To our knowledge, no study has solely focused on the outcomes of staphylococcal PJI in hip hemiarthroplasty (HA), which is a different entity. The surgical setting of a traumatic injury and the characteristics of patients treated with HA, who tend to be older and frequently have more medical comorbidities, may be potential factors behind the reported higher incidence of PJI after HA compared to elective total joint replacement [14,15].

This study aims to retrospectively review the clinical outcomes after staphylococcal prosthetic hip HA infections, based on the results of a level 1 trauma center. We aimed to provide an estimate regarding the incidence of complications and the expected outcomes after different treatment options.

## 2. Materials and Methods

### 2.1. Study Protocol

After Institutional Review Board approval, a retrospective study was performed to evaluate all hemiarthroplasty patients in the electronic medical records, using CPT codes. A total of 2477 HA cases were identified and retrieved from the electronic medical database. All the cases were individually reviewed against our inclusion and exclusion criteria to extract the cases and data relevant to this study. Data collected included patients’ demographics, clinical information, laboratory results, and radiographic evaluation. Detailed information about follow-up and post-operative clinical courses was collected for all cases.

### 2.2. Inclusion Criteria

1. Patients who underwent hemiarthroplasty for a hip fracture, either primarily after trauma or for the management of femoral neck fracture nonunion.
2. Patients diagnosed with postoperative hematoma or postoperative prosthetic joint infection.

### 2.3. Exclusion Criteria

1. Patients with bacterial culture results indicating non-staphylococcal colonization.
2. Patients who had negative bacterial culture results.

### 2.4. Statistical Analysis

Statistical analysis was performed using SPSS software version 25. The mean values, standard deviation, ranges, and proportions were calculated. The *t*-test was used to compare incidences of outcomes. *p*-value above 0.05 was considered insignificant.

## 3. Results

### 3.1. Patients

**Figure 1:**
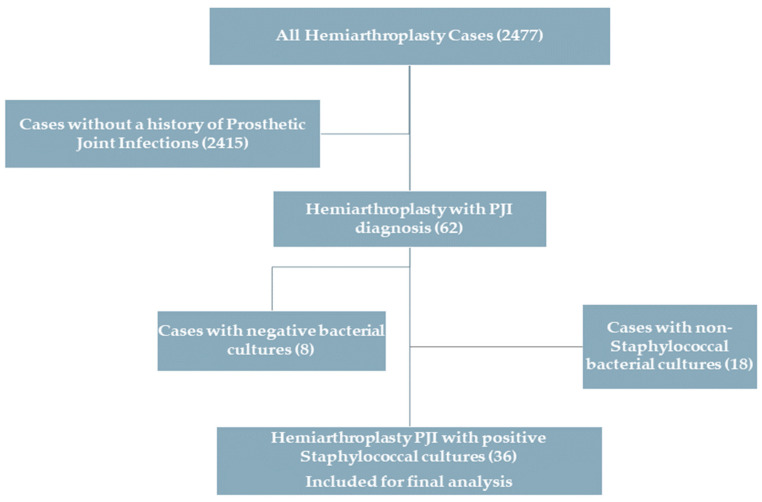
Flowchart of the selection criteria.

Out of the reviewed 2477 hemiarthroplasty cases that could be retrieved from the institutional electronic medical database, a total of 62 cases (2.5%) with prosthetic joint infections requiring surgical intervention were identified. Of these, 26 cases were excluded for not fulfilling the selection criteria, leaving 36 cases (36 patients) for analysis and inclusion in the study (Figure 1).

Of these patients, 26 were female and 10 were male (ratio 2.6:1) with a mean age of 76.5 years (range 52.6–100) at the time of primary HA surgery. Nineteen cases were treated with bipolar, and seventeen were treated with a unipolar hemiarthroplasty. Twenty-five cases were treated with cemented and eleven were treated with cementless stems. A total of 34 cases had significant, frequently combined medical comorbidities in the form of Diabetes mellitus (12 cases), chronic kidney disease (9 cases, with 2 having end-stage renal disease), heart failure (7 cases), hypothyroidism (5 cases), rheumatoid arthritis and chronic epilepsy (2 cases each), morbid obesity (body mass index (BMI) > 40), and dementia and hyperparathyroidism (1 case each). The mean BMI for the patients was 27 (range, 16–40.4). The average follow-up for all the cases was 43.5 months. Twenty patients (55.6%) died at a mean of 20.9 months after the primary HA surgery (range 1.2–99 months).

Regarding the time of presentation, 26 cases presented within 3 months after the index surgery (early PJI) [16], and 10 cases presented more than 3 months (late PJI) after the index surgery (Table 1). The patients presented with two or more of the following concerns: persistent wound drainage, wound sinus, hip pain, prosthesis dislocation, or wound swelling and inflammation. The diagnosis of PJI was established by the treating surgeon, based on the symptoms and signs at presentation. Serial bacterial cultures were obtained from all cases and were proven positive for staphylococcal colonization. Fifteen cases had infections with methicillin-resistant *Staphylococcus aureus* (MRSA) while twenty-one cases had infections with other *Staphylococcus* species. Twenty-five patients had bacterial cultures positive for only *Staphylococcus* and eleven patients had polymicrobial infections with bacterial cultures positive for *Staphylococcus* with other microorganisms. Patients who presented with wound drainage had wound cultures taken preoperatively, and all patients who underwent surgery (35 patients) had serial bacterial wound cultures, or implant sonication that showed staphylococcal colonization. The Gram-stain was used to verify the bacterium morphologically using microscopy, followed by biochemical phenotypic tests (such as the novobiocin, catalase, and coagulase tests) and/or specific culture media (such as blood agar, salty mannitol agar, and DNAse agar) to identify the staphylococcal species [17].

### 3.2. Clinical Outcomes

**Figure 2:**
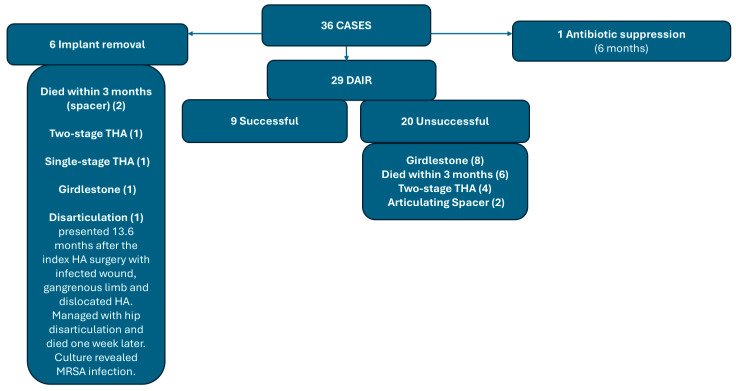
A Flowchart shows the outcomes in all the study cases.

Of the 36 patients included in this study, 35 of these cases were managed with surgical intervention which included one or more of the following: wound debridement and irrigation, the exchange of modular components, the placement of antibiotic spacer, revision to total hip arthroplasty, Girdlestone procedure, or hip disarticulation. In another case, in a 63-year-old male, chronic suppressive antibiotic therapy was utilized without surgical intervention. The most common outcomes were successful DAIR (9, 25%), Girdlestone procedure (9, 25%), and early mortality within 3 months of the primary surgery (8, 22.2%). The overall outcomes in all cases are summarized in Figure 2 and Table 1. The 3-month, 1-year, 3-year, and 5-year mortality rates are 22.2, 30.5, 41.7, and 50%, respectively (Table 1).

### 3.3. DAIR Surgery Outcomes (Twenty-Nine Cases)

Twenty-nine cases underwent at least a single DAIR trial surgery (Figure 2, Table 2). Successful DAIR defined by the clinical and laboratory eradication of infection, and retention of implants without 3-month mortality was achieved in nine cases (31%). Of these nine cases, eight of these infections were early and one was a late acute hematogenous infection [16]. Following successful DAIR, the mean infection-free period for the nine cases was 44.6 months.

Twenty cases underwent unsuccessful DAIR surgery. Of these cases, sixteen presented as early, and four cases presented as late. The outcomes for the unsuccessful DAIR group are presented in Figure 2 and Table 2. The mean time from presentation to DAIR surgery correlated significantly with the success of implant retention, with the cases with unsuccessful DAIR showing more time till surgical intervention (Table 2).

### 3.4. Primary Implant Removal Surgery Outcomes (6 Cases)

Six cases were primarily managed with implant removal at the initial surgical intervention for PJI. These patients included four females and two males with a mean age of 76.3 years. Two of these cases presented with early PJI and four presented with late PJI, with a mean time to presentation of 21.7 weeks (range 2–59) from the index HA. The outcomes for these patients varied. Surgical management included a one- or two-stage revision to THA, Girdlestone procedure, antibiotic spacer, and hip disarticulation (Figure 2).

## 4. Discussion

In this retrospective study evaluating the outcomes after staphylococcal periprosthetic joint infections in hip hemiarthroplasty cases, we found a significantly high incidence of morbidity and mortality.

*Staphylococcus* species is reported to be the causative organism in about two-thirds of PJI cases [5], with *Staphylococcus aureus* alone accounting for about one-third of PJI cases [5,18]. In this study, *Staphylococcus aureus* was responsible for 23 cases of all the 62 PPI cases in our database (37%), and there were 13 further cases caused by other staphylococcal species (21%). When added together, *Staphylococcus* species was responsible for 58% of infected HA cases in our study.

Several studies have reviewed the outcomes of staphylococcal infections in patients undergoing total joint arthroplasty [6,7,8,9,10,11,12,13,18]. However, these studies report collectively on all PJI cases, combining total hip, total knee, shoulder arthroplasty, and hip hemiarthroplasty cases. In hemiarthroplasty, being a different setting from total joint arthroplasty, it has been shown that PPI occurs in an exceptionally higher incidence than elective total hip arthroplasty (1.6–10% vs. 0.2–0.7%, respectively) [14,15]. This is likely related to the patient characteristics and medical comorbidities of patients treated with HA, being older and having more medical issues, as well as the presence of trauma [14,15]. To the best of our knowledge, this is the first study to focus specifically on the outcomes of staphylococcal PJI in HA patients. Our results suggest overall worse outcomes regarding morbidity and mortality.

The management of PPI falls under two broad treatment options, namely, implant retention (debridement, antibiotics, implant retention, i.e., DAIR) or implant removal (IRm). The decision to perform DAIR or IRm in PJI depends on many factors, including the timing of infection, medical history, causative organism, and the host [16,18]. For late presentation cases, IRm with two-stage revision arthroplasty remains the gold standard treatment, despite the increasing evidence that supports single-stage revision in selected cases [19].

Several studies have reported on the outcomes of DAIR surgery in PJI [6,7,8,9,10]. Infections caused by acute hematogenous infections, *Staphylococcus aureus*, trauma cases, early-onset prosthetic joint infection, and patients treated by non-arthroplasty surgeons have a higher reported failure rate [8,20,21]. Byren et al. [8] reported a failure rate of 18% in the first 2.5 years following DAIR for PJI cases. The authors identified *Staphylococcus aureus* as an independent risk factor for DAIR failure. Westhuizen-Bakker et al. [20] reported an overall incidence of failure of 45% in DAIR surgery for late PJI cases. This failure rate increased significantly for the following risk factors: the causative organism of *Staphylococcus aureus* (55% failure rate), hemiarthroplasty, age above 80 years, and rheumatoid arthritis. They concluded these were all independent risk factors for DAIR failure. Bourget-Murray et al. [21] reviewed the predisposing factors and the outcomes of 44 infected HA and found that patients with higher Charlson Morbidity Index score, patients with cardiovascular or peripheral vascular disease, patients with moderate to severe renal disease, and patients with cancers were associated with increased PJI risk. The authors reported a 57.7% failure of DAIR surgery, a 1-year overall mortality of 22.7%, and recommended single-stage revision arthroplasty with cemented components for HA PJI [21]. In our study, the overall failure rate in the 29 cases that underwent DAIR is 69%. It is worth noting that, in our cases, the time from presentation until the first DAIR was found to be almost double in the DAIR failure cases (3.4 days ± 2.1 versus 10.6 days ± 13.7 (*p* = 0.03). We also noted that none of the late PJI cases that received DAIR in our series had successful DAIR outcomes, except one acute hematogenous late PJI case that had a successful DAIR after a 10-month follow-up before the patient’s death from unrelated causes. These results align with the results of Tsang et al. [22], who recommended that DAIR surgery should be performed within 1 week after the presentation as a delayed DAIR after a median of 1 week led to decreased success, with success rates dropping from 72% to 51.8% with delayed DAIR.

As for the results of specific *Staphylococcus* species, several studies on MRSA infections of hip and knee arthroplasty reported successful eradication results as low as 46–50% [23,24,25]. Over the last three decades, MRSA has shown a constantly increasing trend as a causative organism for PJI [26].

Our study reports exceptionally high cumulative mortality rates. While the 3-month mortality in this study was 22.2%, the 1-year, 3-year, and 5-year mortality rates were 30.5%, 41.6%, and 50%, respectively. These numbers are higher than the rates provided in other studies concerning prosthetic joint infections, which ranged from 5 to 10.6% for 1-year mortality [27,28,29], 7.5 to 13.6% for two-year mortality [27,29], and 25.9% for five-year mortality [30]. The higher mortality rates in this study could be attributed to our study only including patients treated with HA as well as the high virulence rate of *Staphylococcus* species. As stated above, the studies that reported mortality outcomes following PJI combined elective total joint arthroplasty patients, who tend to be relatively young and healthy, with hemiarthroplasty cases. In addition, other studies did not distinguish between the mortality rates for staphylococcal versus non-staphylococcal infections, despite the high discrepancy in virulence. In any case, the mortality after periprosthetic infections, in general, remains significantly high. Zmistowski et al. [29] even compared the relative survival rate at five years after prosthetic joint infection onset, which was 73.9% in their study, to one of the five most common cancers, namely prostate cancer (99%), melanoma (91%), breast cancer (89%), colorectal cancer (64%), and lung cancer (16% 5-year mortality rate). Although the high mortality rate associated with staphylococcal PJI in our study may be confounded by factors such as patient age, underlying medical conditions, or the inherent mortality risk of hip fractures, a comparison of the cumulative 1-year mortality in our study (30.5%) with the reported 1-year mortality rates for hip hemiarthroplasty following hip fractures (18.5–28%) [30,31] suggests that mortality following PJI, and staphylococcal PJI in particular, appears to be higher.

The results of this study demonstrate that staphylococcal PJI is a significant complication that is associated with very high morbidity and mortality in hip hemiarthroplasty cases. With the increasing trends towards MRSA infections and the wide utilization of hip hemiarthroplasty for femoral neck fractures in elderly and low-demand patients [1,32,33,34], these results could provide evidence for the importance of proper patient optimization and careful infection prevention strategies [35,36], including preoperative staphylococcal decolonization [37] to help prevent such severe complications.

This study has several limitations. The small number of cases is a major limitation. The retrospective nature of the study, which spans a long period, is also a major limitation that could have specifically caused discrepancies in the diagnostic procedure of PJI and the protocols used for identifying the staphylococcal species. However, we aimed to provide a reference regarding the outcomes of specific microorganisms in a specific group of patients. Ideally, a multi-center study on a larger number of cases would be appropriate to generate stronger evidence.

## 5. Conclusions

Staphylococcal prosthetic joint infection of hip hemiarthroplasty is a significant complication that is associated with a low success of implant retention surgery (31%), very high morbidity, and high cumulative mortality.

## Figures and Tables

**Table 1:** Early versus late cases.

	Early Infection (Presented Within 3 Months After HA Surgery) (*n* = 26)	Late Infection (Presented After 3 Months Post-HA Surgery) (*n* = 10)	Total Cases (36)
Time of presentation (mean, range) weeks	3.45 weeks (1–10)	53.4 weeks (15.7–217.3)	17.7 weeks (1–217.3)
Age	79 (52.6–98.8)	69.7 (53.5–100)	76.5 years (52.6–100)
BMI	27 (16.2–39)	27 (16–40.4)	27 (range, 16–40.4)
Causative organism	MRSA (11) *Staphylococcus aureus* (6) Coagulase-negative staphylococci (2) *Staphylococcus hemolyticus* (1) *Staphylococcus epidermidis* (1) Other staphylococci (5)	MRSA (4) *Staphylococcus aureus* (2) Other staphylococci (4)	MRSA (15) Non-MRSA (8) Other staphylococci (13)
Outcome	DAIR: 24 cases: - Successful DAIR, 8 cases (33.3%) - Unsuccessful DAIR, 16 cases (66.7%): 7 Girdlestone; 6 died within 3 months; 3 two-stage total hip arthroplasties (THA); Implant removal, 2 spacer cases, both died within 3 months.	DAIR: 5 cases - Successful DAIR, 1 case, acute hematogenous (20%): - Unsuccessful DAIR, 4 cases (80%): 2 spacer; 1 Girdlestone; 1 two-stage THA. Implant removal, 4 cases: - 1 single-stage THA; - 1 two-stage THA; - 1 Girdlestone - 1 disarticulation 1 Antibiotic suppression	Successful DAIR 9 (25%); Girdlestone procedure 9 (25%); Died within 3 months from the index HA surgery 8 (22.2%). Two-stage THA 5 (13.9%); Spacer 2 (5.56%); Single-stage THA 1 (2.78%); Antibiotic suppression 1 (2.78%); Hip disarticulation 1 (2.78%); Mortality at: 3 months: 8 cases (22.2%) 1 year (+3 cases): 11 (30.5%) 3 years (+4 cases): 15 (41.7%) 5 years (+3 cases): 18 (50%)
Successful DAIR	8/24 (33.3%)	1/5 (20%)	

**Table 2:** Successful versus unsuccessful DAIR cases (29 cases).

	Successful DAIR	Unsuccessful DAIR
**Number**	9 (31%)	20 (69%)
**Age**	78.8 (59.7–100)	76.3 (52.6–98.9)
**Time from HA till presentation**	1 late acute hematogenous (11.1%), 8 early (88.9%)	4 late (20%), 16 early (80%)
**Time from presentation till DAIR surgery**	3.4 days ± 2.1 (range, 1–8 days)	10.6 days ± 13.7 (range, 1–62)
	***p* = 0.03 (Welch *t*-test)** **(statistically significant**)	
**Type of implant**	Cemented 8 Uncemented 1	Cemented 14 Uncemented 6
**Number of DAIR trials**	1 (6 cases) 2 (3 cases)	1 (5 cases) 2 (11 cases) 3 (4 cases)
**Outcome**	The mean infection-free follow-up was 44.6 months	Girdlestone procedure: 8 cases Died within 3 months of index HA: 6 cases Two-stage revision THA: 4 cases Spacer: 2 cases
**Causative organism**	Staphylococcus aureus (4) Coagulase-positive staphylococci (2) MRSA (1) Other Staphylococcus strain (2)	MRSA (11) *Staphylococcus aureus* (3) *Staphylococcus epidermidis* (1) *Staphylococcus hemolyticus* (1) Another *Staphylococcus* strain (4)

## Data Availability

All data generated or analyzed during this study are included in this published article.
